# EVALUATION OF A RADON AIR MONITOR IN THE MEASUREMENT OF RADON CONCENTRATION IN WATER IN COMPARISON WITH A LIQUID SCINTILLATION COUNTER

**DOI:** 10.1093/rpd/ncz070

**Published:** 2019-04-30

**Authors:** Shoko Higuchi, Yuuki Kamishiro, Maki Ishihara, Yumi Yasuoka, Yasunori Mori, Masahiro Hosoda, Kazuki Iwaoka, Shinji Tokonami, Rikiya Takahashi, Miroslaw Janik, Jun Muto, Hiroyuki Nagahama, Takahiro Mukai

**Affiliations:** 1 Kobe Pharmaceutical University, Kobe City, Hyogo, Japan; 2 Mie Prefecture Health and Environment Research Institute, Yokkaichi City, Mie, Japan; 3 Hirosaki University, Hirosaki City, Aomori, Japan; 4 National Institutes for Quantum and Radiological Science and Technology, Chiba City, Chiba, Japan; 5 Hirosaki University Hospital, Hirosaki City, Aomori, Japan; 6 Department of Geoenvironmental Science, Graduate School of Science, Tohoku University Sendai City, Miyagi, Japan

## Abstract

The World Health Organisation (WHO) recommends that the concentration of radon in water should be no more than 100 kBq m^−3^ (100 BqL^−1^) and the Codex Alimentarius Commission states that the limit of quantification (LOQ) of a method should be no more than one-fifth of this value. In this study, a degassing method with an RAD7 device was used to measure radon concentrations in water, compared to a liquid scintillation counter (LSC) method used as the reference, to investigate whether the numerical value of the LOQ of this method was more than 1/5 (20 kBq m^–3^) of 100 kBq m^–3^. The degassing method with leak prevention was shown to reach a target value of 20 kBq m^−3^ or less under a relative humidity of 6% or lower in the chamber of the RAD7 device. Accordingly, the RAD7 degassing method with leak prevention can be used to accurately measure radon concentrations in water within the guidance level set out by the WHO.

## INTRODUCTION

The WHO guidelines for radon (^222^Rn) concentration in water^(1)^ suggest that acceptable levels should be no more than 100 kBq m^–3^, and the Codex Alimentarius Commission states that the numerical value of the limit of quantification (LOQ) of the measuring method should be no more than one-fifth of the specified guidance level^(2)^. In other words, the numerical value of LOQ should be no more than 20 kBq m^–3^, based on the official WHO guidance level of 100 kBq m^–3^. Radon concentration in water is generally measured using the liquid scintillation counting (LSC) method. However, the scintillation cocktail (organic solvent) used in the LSC method is difficult to transport, which limits the performing LSC method to local sites. An alternative LSC method that can be transported to many areas of the world is highly desired. When the measuring conditions prohibit the performing LSC method, we investigate whether standard LSC can be replaced by the degassing method.

When using a RAD7 (DURRIDGE Company Inc., USA) radon air monitor connected to a RAD7 H_2_O accessory (RAD7 degassing method) as one of the degassing methods, radon concentrations in water were measured. Some previous studies^(3, 4)^ reported that they measured radon in water when RAD7’s indicator showed the relative humidity should be 10% or below as instructed in the RAD7’s manual^(5)^. And Stojković *et al.*^(6)^ reported that the radon concentrations measured using RAD7 were lower than those measured using the LSC method when the relative humidity indicator of RAD7 showed 8% or below during the measurements. On the contrary, Somashekar and Ravikumar^(7)^ indicated that it is also necessary to maintain the relative humidity indicator of RAD7 at 6% or below. Lee *et al.*^(8)^ reported that radon concentrations measured with RAD7 matched those measured using the LSC method, although they only detailed one piece of data, at a value of around 47 kBq m^–3^, and did not specify whether the numerical value for the LOQ of the RAD7 degassing method was one-fifth of the specified guidance level (20 kBq m^–3^).

In this study, we investigated whether the numerical value for the LOQ of the RAD7 degassing method is one-fifth (20 kBq m^–3^) that of the specified guidance level (100 kBq m^–3^). The RAD7 degassing method was assessed using the LSC method in reference to the performance specifications for systems designed to measure radon gas in air^(9, 10)^. The following criteria were adopted: the relative standard deviation percentage (RSD%) of each set of measurements should be no greater than 15%, as the precision, and the individual percentage error (IPE%) should be between −25% and 25%, as the accuracy (i.e. the difference between the two methods should be negligible). The IPE% means the range of the relative percentage difference between the two radon monitors.

## METHODS

The RAD7 device was calibrated in a radon standard chamber located at the National Institute of Radiological Science (NIRS), and the calibration constant of RAD7 was determined to be *F*_R_ = 0.909^(11)^. The background value of the device was measured. The monitor of the device displayed a value of zero because nothing was detected; therefore, the background value was considered zero.

The LSC method for measuring the radon concentration in water was used as a reference method in this study. The method are expressed by the subscripts R (the RAD7 degassing method) and S (the LSC method).

### Validation of the RAD7 degassing method by measuring radon concentrations in water

To test whether the LOQ of the RAD7 degassing method is below 20 kBq m^–3^, we compared the RAD7 degassing method with the LSC method (reference method). The LSC method is a standardised method used globally. Multiple samples were measured from the same water source. One sample was used to test the RAD7 degassing method and three reference samples were used for the LSC method. It is difficult to measure multiple samples using RAD7 degassing method, since it takes several hours for drying the RAD7 chamber completely.

In the RAD7 degassing method, the radon concentration in water was measured with RAD7 equipment using the RAD H_2_O accessory. Before starting the RAD7 degassing method, the chamber of the RAD7 device was checked to ensure that it was dry (i.e. the relative humidity inside was below 5%) and free of radon. One 250 ml sample of water was used as the test sample. And then, the water sample, a 35 g of desiccant, a filter and RAD7 were placed in a closed loop^(5)^. In case A, the measurements were performed while preventing leakage from the RAD H_2_O aerator cap and the drying tube. The leaks were blocked by tape with superior gas tightness and adhesion properties. In case B, the measurements were performed in the original configuration with no leak prevention. The pump to aerate the sample and transfer the radon to RAD7 was operated for 5 min. The radon concentration was counted four times, each time for a period of 5 min. Using a radon concentration *C*_Ri_ (kBq m^–3^) of the original display value measured with RAD7, the radon concentration *C*_R_ (kBq m^−3^) was obtained using the following equation:

(1) $$C_{R}=C_{Ri}/F_{R}$$

In the LSC method, three reference samples were prepared. The water sample (10 mL) was added to a highly efficient mineral oil scintillation cocktail (10 mL; PerkinElmer Inc., USA) in a 20 mL glass LSC vial (PerkinElmer Inc., USA). The radon concentrations *C*_S_ (kBq m^−3^) in the samples were measured by the integral counting method in the LSC method (2300TR, PerkinElmer Inc., USA) for 60 min^(12)^.

## RESULTS AND DISCUSSION

### Assessing the RAD7 degassing method for the measurement of radon concentrations in water

Multiple samples were tested from the same water source. When the range of radon concentrations in water was between 9.0 and 89 kBq m^–3^, we simultaneously measured using the values of RAD7 degassing method (mean ± standard deviation=*C*_R_ ± σ_R_) kBq m^−3^ (four sets of measurements) and the LSC method (mean ± standard deviation=*C*_S_ ± σ_S_) kBq m^−3^ (three sets of measurements).

#### Precision

**Figure 1.**
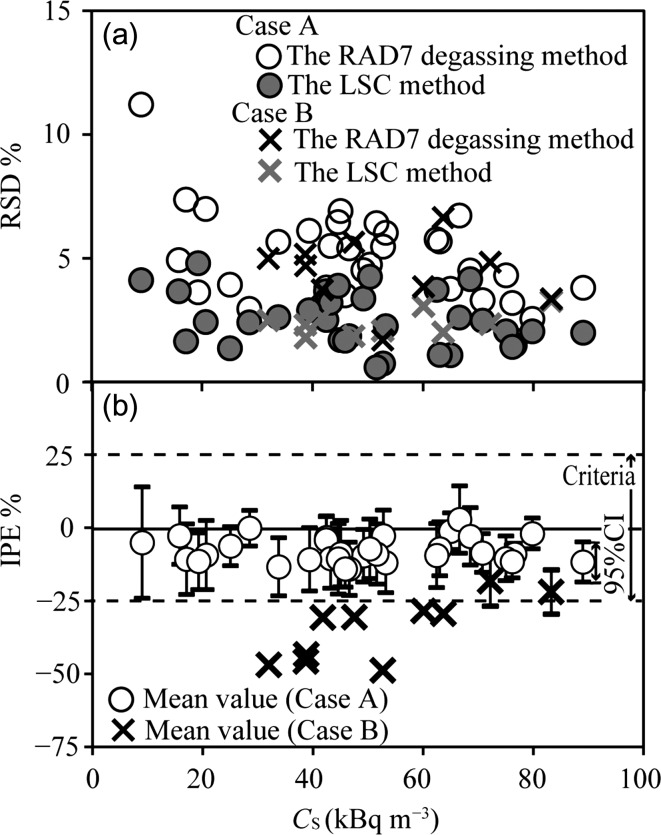
Assessment of the RAD7 degassing method. (a) Assessment of the precision based on RSD%. (b) Assessment of the accuracy based on IPE%. For points lacking 95% CI error bars, the average IPE% was below −25%.

First, we determined the lower quantitative limit of radon concentration of the RAD7 degassing method (or the LSC method) that satisfies the optimal precision requirements. The RSD% values of the RAD7 and LSC methods were found to be (100 σ_R_/*C*_S_)% and (100 σ_S_/*C*_S_)%, respectively. Figure 1a shows the relation between the *C*_S_ and RSD% values of RAD7 (or the LSC method) used to test the optimal precision in cases A and B. All RSD% values of RAD7 (or the LSC method) in both cases were below 15%, all of which were determined to satisfy the optimal precision requirements.

#### Accuracy

Next, we determined the lower quantitative limit of radon concentration of the RAD7 method (or the LSC reference method) that satisfies the optimal accuracy requirements, using the IPE%. The IEP% was expressed as mean and standard deviation (*D* ± σ_D_)%. We calculated the lowest IPE% values of the RAD7 method that satisfy the optimal precision requirements. The IPE% between a pair of measurements simultaneously recorded by the detectors can be calculated using Eq. (2). When the IPE% value is between −25% and 25%, the radon concentration satisfies the optimal precision requirements, within control:

(2) $$D\pm \sigma_{D}=100\left\{\left(C_{R}\pm \sigma_{R}\right)-\left(C_{S}\pm \sigma_{S}\right)\right\}/\left(C_{S}\pm \sigma_{S}\right)$$

When validating the measurement of radon concentrations in water using the RAD7 degassing method, the 95% confidence interval (95% CI) was used. The 95% CI, which includes the upper 95% CI and lower 95% CI of IPE%, is given in the following equation:

(3) $$D-k\sigma_{D}\sqrt{1/n}≦95\%\mathrm{CI}≦D+k\sigma_{D}\sqrt{1/n}$$

where *n* (*n* = 4) represents the number of measurements using the RAD7 degassing method and *k* (*k* = 3.18) represents the *t*-value from Student’s the t-distribution (two tailed) table

Figure 1b shows the relation between the *C*_S_ and IPE% values of the RAD7 method used to determine the accuracy precision. In case A when the radon concentration in the water was more than 9 kBq m^–3^, all 95% CI values of the IPE% of the RAD7 method (error bars with open circles in Figure 1b) were sufficiently ensured to lie between 25% and –25%. The relative humidity in RAD7 was ≤ 6%. Therefore, we determined that the RAD7 degassing method with leak prevention can be performed in the optimal accuracy when radon concentration in the water was more than 9 kBq m^–3^. The LOQ of the RAD7 degassing method is below 20 kBq m^–3^.

On the other hand, the RAD7 method failed in case B, because the lower 95% CI values (error bars with cross marks in Figure 1b) or the average values were below –25%. The relative humidity in RAD7 was approximately 10%. It was found that air leakage from the upper point of the drying tube increased the relative humidity in RAD7 to above 6%. We also identified air leaks from the RAD H_2_O aerator cap and the drying tub. Comparisons with the LSC method confirmed the need for leak prevention in the degassing method.

However, it was not possible to completely prevent radon leakage, which is one of the disadvantages of the proposed degassing method, since all IPE% were below 0 (the equivalent) in Figure 1b. From the above results, we conclude that when the conditions are not favourable to conduct LSC, the degassing method with leak prevention can be used.
